# Developing a Cloud-Based Air Quality Monitoring Platform Using Low-Cost Sensors

**DOI:** 10.3390/s24030945

**Published:** 2024-02-01

**Authors:** Abdul Samad, Joschka Kieser, Ioannis Chourdakis, Ulrich Vogt

**Affiliations:** Institute of Combustion and Power Plant Technology (IFK), Department of Flue Gas Cleaning and Air Quality Control, University of Stuttgart, Pfaffenwaldring 23, 70569 Stuttgart, Germany

**Keywords:** low-cost sensors, gas sensors, PM sensors, air quality sensors, electrochemical low-cost sensors, air pollutants, air quality monitoring

## Abstract

Conventional air quality monitoring has been traditionally carried out in a few fixed places with expensive measuring equipment. This results in sparse spatial air quality data, which do not represent the real air quality of an entire area, e.g., when hot spots are missing. To obtain air quality data with higher spatial and temporal resolution, this research focused on developing a low-cost network of cloud-based air quality measurement platforms. These platforms should be able to measure air quality parameters including particulate matter (PM10, PM2.5, PM1) as well as gases like NO, NO_2_, O_3_, and CO, air temperature, and relative humidity. These parameters were measured every second and transmitted to a cloud server every minute on average. The platform developed during this research used one main computer to read the sensor data, process it, and store it in the cloud. Three prototypes were tested in the field: two of them at a busy traffic site in Stuttgart, Marienplatz and one at a remote site, Ötisheim, where measurements were performed near busy railroad tracks. The developed platform had around 1500 € in materials costs for one Air Quality Sensor Node and proved to be robust during the measurement phase. The notion of employing a Proportional–Integral–Derivative (PID) controller for the efficient working of a dryer that is used to reduce the negative effect of meteorological parameters such as air temperature and relative humidity on the measurement results was also pursued. This is seen as one way to improve the quality of data captured by low-cost sensors.

## 1. Introduction

It has been scientific consensus for some time that polluted air has a significant negative impact on human health and the environment. This problem affects low-, middle-, and high-income countries equally. Exposure effects on human health range from minor upper respiratory irritation to chronic respiratory and heart diseases like Chronic Obstructive Pulmonary Disease (COPD). Even an increased risk of lung cancer is associated with long-term exposure to polluted air [1,2]. Some air pollutants like black carbon, which can be found as a part of the particulate matter (PM) load and ground-level ozone (O_3_), are not only negatively affecting human health but are also responsible for near-term warming of the environment. Therefore, reducing air pollution can have a mitigating effect on climate change [2]. Air pollution originates from two major sources, natural emissions and anthropogenic emissions. Natural emission sources include volcanic eruptions and forest fires. The main cause of human-made emissions is combustion processes. Whether for energy generation, industrial production, or transportation, air pollutants are released as soon as fossil fuels or biomass is burned. Even indoors, combustion processes from, for example, heating or cooking can be responsible for poor air quality [2].

Some of the major pollutants worth observing as described by the World Health Organization (WHO) are PM, NO, NO_2_, O_3_, CO, and SO_2_. Recently, new recommendations were published regarding the maximum tolerable concentrations of these pollutants in the ambient air [3]. These new guidelines are even more stringent than those previously postulated in the year 2005. This is due to improved measurement methods as well as new insights into the health effects of air pollutants as well as their impact on animals and plants.

Governments and legislators worldwide have a high influence on improving air quality. However, for these entities to measure the impact of their actions on air pollution, accurate spatial and temporal monitoring data of air pollutants are needed. This is especially important in highly populated urban environments where the highest pollutant concentrations can be observed [3]. To achieve this, many cities have deployed a small number of very accurate but expensive air quality monitoring stations.

Several official static monitoring stations located in Stuttgart concluded an improvement in air quality over time from 2005 to 2020 [4]. These measurements only took place in two spots and might not capture the entire air quality in the city. This is a good example where a dense network of low-cost air quality sensors could obtain sufficient spatio-temporal resolution to expose potential hot spots of air pollution. In other countries like Israel [5] or the United Kingdom similar sensor networks have already been implemented.

### 1.1. Previous Studies

**Figure 1:**
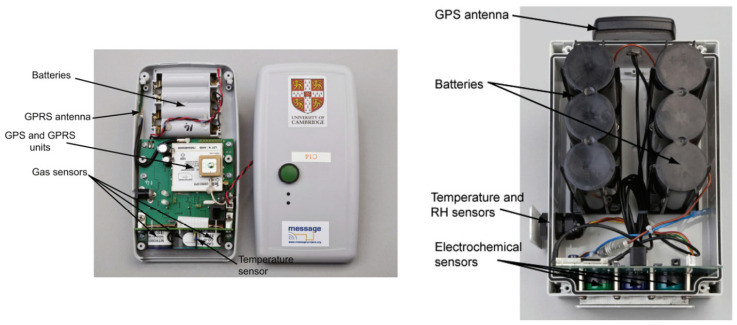
An overview of mobile WSN (**left**) and stationary WSN (**right**) [6].

Several low-cost air quality sensors with networking capability have already been developed. In 2013, e.g., Mead et al. [6] developed measurement platforms for ambient air using Alphasense electrochemical gas sensors. These clearly showed that the sensors, which were designed for the parts-per-million (ppm) range, can also be used in the parts-per-billion (ppb) range with the right adequate calibration. This higher resolution is necessary to detect the pollution in the ambient air. The resulting battery-powered Wireless Sensor Networks (WSNs) were equipped with a Global Positioning System (GPS) for localization and a cellular General Packet Radio Service (GPRS) system for transmitting the measurement data to a central server. Lightweight nodes were developed for mobile measurements as can be seen in Figure 1. But there were also stationary nodes equipped with heavy lead batteries for measurement campaigns lasting several months. To prove sensor reproducibility, multiple pairs of mobile WSNs were collocated while moving through central London. This experiment resulted in a mean squared error of R^2^ ≅ 0.95 for the gases NO, NO_2_, and CO indicating a strong correspondence. The continuation of this research led to a network of low-cost WSNs deployed at the London Heathrow Airport by Popoola et al. [7]. From a total of 50 available WSNs, 28 were positioned at different sites surrounding the airport. In addition to the gas sensors of their predecessors, these WSNs have been equipped with a PM sensor, an anemometer, and temperature/humidity sensors.

**Figure 2:**
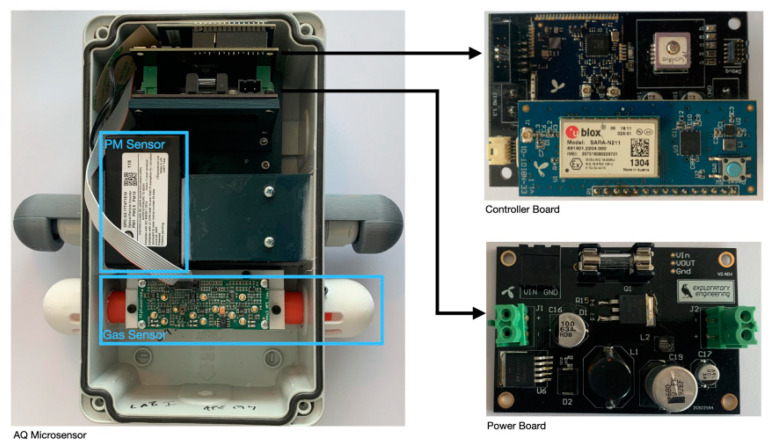
An overview of Trondheim stationary WSN [8].

A more recent network of 25 low-cost air quality sensors was established in Trondheim, Norway in 2020. The paper by Veiga et al. [8] appeared in 2021, and the hardware overview of a WSN from it can be seen in Figure 2. It features the same series of Alphasense electrochemical gas sensors that were also utilized by Popoola et al. [7]. Both studies employ a passive measurement air sampling approach on the membranes of these gas sensors. To measure PM, an Optical Particle Counter (OPC) sensor Alphasense OPC-N3 was chosen. Without, for example, a heater to dehumidify the measured air, the PM concentration will be overestimated at high ambient humidity levels. This effect was largely eliminated in the study by using a random forest regressor. However, this was only possible after incorporating local weather data in the input dataset and had to be performed for each sensor. To train and test the model, data from a local official air quality measurement station was utilized. This was possible due to colocation with a low-cost WSN. Another two low-cost WSNs were collocated in the same way to test for reproducibility. The geolocation was ascertained using a GPS receiver and the collected data were transferred to the cloud using Narrowband Internet of Things (NB-IoT), a cellular Low-Power Wide-Area Network (LPWAN) technology transmitting on the same frequencies as Fourth-Generation Long-Term Evolution (4G/LTE).

The research previously conducted at the University of Stuttgart regarding low-cost air quality sensors focused on the factors that affected the data quality such as relative humidity, air temperature, etc. A low-cost dryer was proposed to be a solution to improve the data quality of the low-cost sensors [9,10]. Nevertheless, improvement was required for the low-cost dryer operation. Also, the two low-cost systems (PM and gas) were in separate housings in previous studies. To use both systems, an upgrade was required for the processing unit as well as the mechanical system.

### 1.2. Objectives

The research described in this paper aimed to develop and validate a low-cost hardware and software platform for distributed air quality measurements. The air quality parameters to be measured consisted of the gases NO, NO_2_, O_3_, and CO as well as PM10, PM2.5, and PM1. The ambient temperature and humidity were measured as well. To support mobile measurement campaigns, a localization system was implemented that recorded the coordinates of each sensor data point. All these measurements need to be processed on each node and stored locally for redundancy. Likewise, the data must be transferred cyclically to the cloud part of the platform utilizing a suitable wireless technology.

The second objective of this research was the development and implementation of a control loop for the inlet dryer used for the dehumidification of the air sampled by the PM and gas sensors. To achieve this, the low-cost heater design previously developed at the Department of Flue Gas Cleaning and Air Quality Control at the University of Stuttgart [9,10] was optimized.

## 2. Methodology

### 2.1. Design Considerations

To find the best solution for the entire system, it is first split into different subsystems. For these subsystems, different solutions can then be compared, and the most suitable solution can be selected. To compare similar subsystem implementations, the concept of morphological charts is used. It allows grading of similar implementations against various functions or problems to find a score of how well the solution matches the requirements. In the next step, every function or problem can be weighted differently to further improve the selection, which results in a weighted score. This score, with 1 being the lowest and 5 being the highest, denotes the suitability of the implementation and will be used to decide what will be used in the final product.

In this research, the system is split into the subsystems: processing unit, wireless interface, sensors, and dryer. Only the wireless interface and processing unit will be selected via a morphological chart as the other two subsystems are mostly predefined.

#### 2.1.1. Processing Unit

To select which processing unit is suited best to fulfill the requirements, a microcontroller, embedded system, and industrial computer are compared in Table 1.

Considering the requirement that the system must be able to provide data processing and storage, the processing of data is weighted at 30%. While the industrial computer performs the best in terms of processing speed, an embedded system can almost reach the same performance. The microcontroller is graded worst as it runs much slower and cannot handle multiple tasks at the same time comparatively. In terms of hardware interfacing capability, which is weighted at 30%, the microcontroller is much more suitable than its competitors as it features General-Purpose Input/Output (GPIO) pins, a multitude of interrupts allowing for real-time applications, and even built-in Analog-to-Digital Converters (ADCs). Most of this functionality is also present in an embedded system, while some features like ADCs or real-time capability are missing. The worst performance in terms of hardware interfacing can be expected from an industrial computer as no GPIOs are present and an additional microcontroller would have to be utilized to enable sensor interaction. The microcontroller also requires the smallest amount of power during operation, with the industrial computer operating at the highest power level. Finally, returning to data processing and storage requirements, the storage of sensor data is considered at a weight of 20%. While a storage medium like a hard drive or Solid-State Drive (SSD) can easily be connected to an industrial computer or embedded system, the microcontroller can only interface with lower-quality flash storage like Secure Digital (SD) cards, giving it the lowest score on storage capability. All in all, the embedded system receives the score in both the weighted and average categories and is thus chosen as the solution for this subsystem. The embedded system that has been selected for the Air Quality Sensor Node (AQSN) prototype implementation of this research study was a Raspberry Pi 4.

#### 2.1.2. Wireless Network Interface

To select which wireless network interface is suited best to match the requirements, the previously introduced wireless interfaces LoRaWAN, Sigfox, and a cellular connection are compared in Table 2.

To fulfill the requirements that the system must be able to provide a long-distance wireless network interface and must be able to send the measured data to the cloud utilizing wireless technology, the data rate and range of the wireless technology are compared and weighted quite high at 20% and 30%, respectively. In this regard, the cellular connection performs the best as much higher data rates can be reached, and the network coverage is far superior to the other two. The data privacy and security concerns while using cloud-based data storage were considered before using it. A possibility to protect the data is to use encryption security tools. This can prevent unauthorized cloud network access. By using VPNs and other encryption tools, the IP address can be masked to conceal cloud traffic and network activity. The system requirements of determining the current location and time were also considered but only resulted in a weight of 10% for the localization of the AQSN and the ability to determine the current time. As most cellular modems come with a built-in GPS receiver, and LoRaWAN or Sigfox only rely on a triangulated localization approach, the cellular connection is the best option in this regard as well. Additionally, the ability to fetch the time accurately is only possible with a cellular connection utilizing, for example, the Network Time Protocol (NTP). Finally, the power consumption of the technology was considered as per the requirement that the system must be able to operate with 230 V AC and is weighted at 20%. Low power usage is the main application scenario for LoRaWAN and Sigfox as they only require very low power to establish a wireless connection. A cellular connection on the other hand requires much higher amounts of power and consequently has the lowest score for this function. In the last point of comparison, i.e., the running costs, LoRaWAN comes off best as it is the only technology that works free of charge with a privately owned gateway. Both Sigfox and a cellular connection require a contract with monthly running costs and are therefore scored poorly. Nevertheless, the cellular connection concludes with the best average and weighted value and was thus used for the implementation of the subsystem wireless network interface. The cellular modem that was selected for the prototype implementation is a Waveshare SIM7600E with built-in GPS.

#### 2.1.3. Sensors

To fulfill the requirements that the system must entail the capability of measuring ambient air concentrations of PM and the gases NO, NO_2_, O_3_, and CO, the same Alphasense sensors (OPC-N3 for PM, CO-B4 for CO gas, NO-B4 for NO gas, NO2-B43F NO_2_ gas, and OX-B431 for O_3_ gas) that have previously been studied at IFK will be reused. The sensors require minimal maintenance as they can be used continuously for around 2 years, and after that, they should be replaced [9,10]. Additionally, to satisfy the requirement that the system must be able to measure ambient temperature and relative humidity, an SHT30 encapsulated in a sintered metal mesh was implemented.

#### 2.1.4. Dryer

The requirement of eliminating the influence of meteorological parameters on the measurement was fulfilled by operating a controlled dehumidification of the measurement air by implementing a low-cost dryer made from resistive wire wrapped around a straight metal tube. This dryer design has previously been implemented and tested by researchers at the Department of Flue Gas Cleaning and Air Quality Control at the University of Stuttgart. The effect of meteorological parameters is significantly reduced by using the dryer, as proved in the previous research by the authors [9,10].

The resistance wire used for the heater coil has a resistance of 1.73 Ω/m. Given an approximate length of 4.5 m, this results in a resistance of R_dryer_ = 7.8 Ω. Considering a dryer voltage of 12 V, ohms law, and the power law:

I_dryer_ = U_dryer_/R_dryer_ ≈ 1.54 A (1)

P_dryer_ = U_dryer_ × I_dryer_ ≈ 18.5 W (2)

This result is congruent with real-world measurements.

Control systems try to externally influence dynamic systems to achieve certain desired states within the system. These states are most commonly represented by observable system values. To measure and influence system states in real-world dynamic systems, sensors and actuators are commonly used. Although technically any dynamic system can be used as a controller, in practice an additive combination of proportional, integral, and differential elements is often used [11]. As it is also required to control the dehumidification process, a PID controller was selected to control the heater coil of the dryer. As previously discussed in the background on the optical PM sensors, the effects of concentration overestimation start at around 50 to 60% relative humidity. This value was selected as a setpoint for the proposed PID controller based on previous studies [12,13,14,15,16].

### 2.2. Node Platform Implementation

#### 2.2.1. Mechanical System

**Figure 3:**
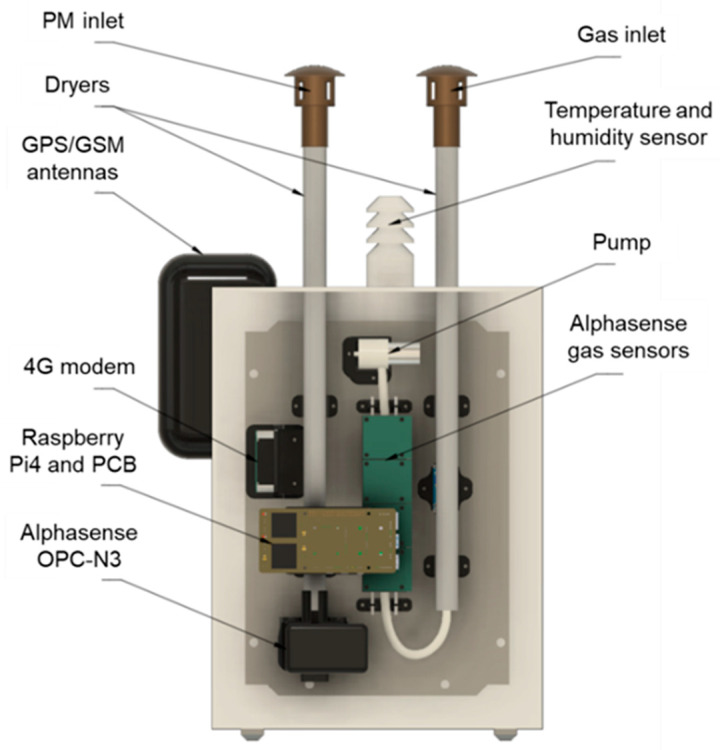
AQSN mechanical design overview.

To fulfill the requirement that the system must shield the electronics from weather, all of the parts that make up an AQSN were housed inside a switching cabinet made from fiberglass. The structure is illustrated in Figure 3. Two separate heaters were used for PM sensor air and gas sensor air dehumidification. While the OPC-N3 includes a fan, a pump is used to actively sample the air from the gas inlet. In the bottom of the switching cabinet, a small hole with a diameter of 4 mm allows air from both the OPC-N3 and the gas pump to leave the housing to prevent pressure build-up inside the enclosure.

The airflow of the gas pump was adjusted to match the flow of the OPC-N3. This way, both heaters can be controlled from the humidity measured at the OPC-N3 and powered in parallel fashion. The dehumidification of measurement air should then be approximately the same for both air pathways.

**Figure 4:**
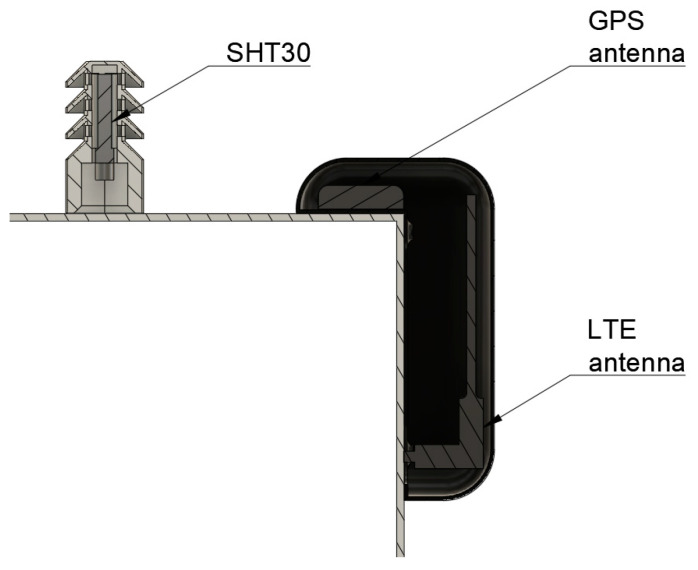
AQSN antenna cut.

To keep rain and dirt from entering the PM or gas inlet, 3D-printed hats have been implemented. They are coated with a copper-based antistatic coating preventing particles from statically charging and sticking to them. The already waterproof SHT30 was also encased inside a 3D-printed hat to keep direct sunlight from influencing the measurements. Figure 4 reveals the SHT30 sensor mounted inside the hat. It also shows how the GPS and LTE antennas are encapsulated inside another 3D printed part, to protect them from the weather and still allow signal reception.

#### 2.2.2. Electronic System

**Figure 5:**
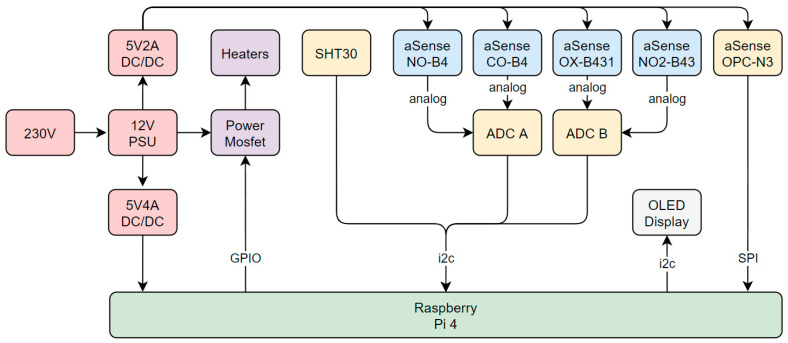
Electronics overview.

The electronic system has a role in fulfilling all the requirements mentioned before and enables the firmware to interface with the hardware. Since a Raspberry Pi was selected as the embedded system, it had to be connected to the sensors and be able to control the dryers. Figure 5 outlines the connections between the Raspberry Pi and the rest of the system.

To begin with, a 12 V power supply runs off 230 V AC. These 12 V were converted to 5 V required for the Raspberry Pi by a DC/DC converter. Another galvanically isolated DC/DC converter was used to supply the Alphasense gas sensors with their required 5 V since insufficient supply voltage stability has a negative effect on measurement quality. While the OPC-N3 was connected directly to the Raspberry Pi via a Serial Peripheral Interface (SPI) bus operating at a frequency of 500 kHz, the analog signal from the Alphasense gas sensors could not be connected directly. To read the gas sensors, two ADS1115 16-bit ADCs were used. After the digitization of the analog signals, they could read into the Raspberry Pi over the I2C bus. The SHT30 temperature and humidity sensor was connected to the same I2C bus. As it does not need a perfectly stable supply voltage, it was powered by the Raspberry Pi. The two low-cost dryers were connected in parallel and driven at 12 V. Since the Raspberry Pi could not switch 36 W at 12 V directly, an optically isolated power Metal-Oxide-Semiconductor Field-Effect Transistor (MOSFET) was used to allow the low-power GPIO to switch the high-power dryer current. By using Pulse Width Modulation (PWM), the fast on and off switching of the MOSFET, an almost step-less control of the dryer power was achieved. The last part shown in Figure 5 is a small Organic Light-Emitting Diode (OLED) display. It was connected to the Raspberry Pi via another I2C interface as it required a higher data rate and frequency than the sensors.

#### 2.2.3. Firmware

Since the Raspberry Pi features a 64-bit ARM processor [17], a full operating system like Linux can be executed. For the Raspberry Pi inside every AQSN, balenaOS [18] based on Yocto Linux was selected. The firmware was implemented in the Python programming language and runs inside a docker container within the operating system. The code developed during this research is open-source and available on GitHub [19].

**Figure 6:**
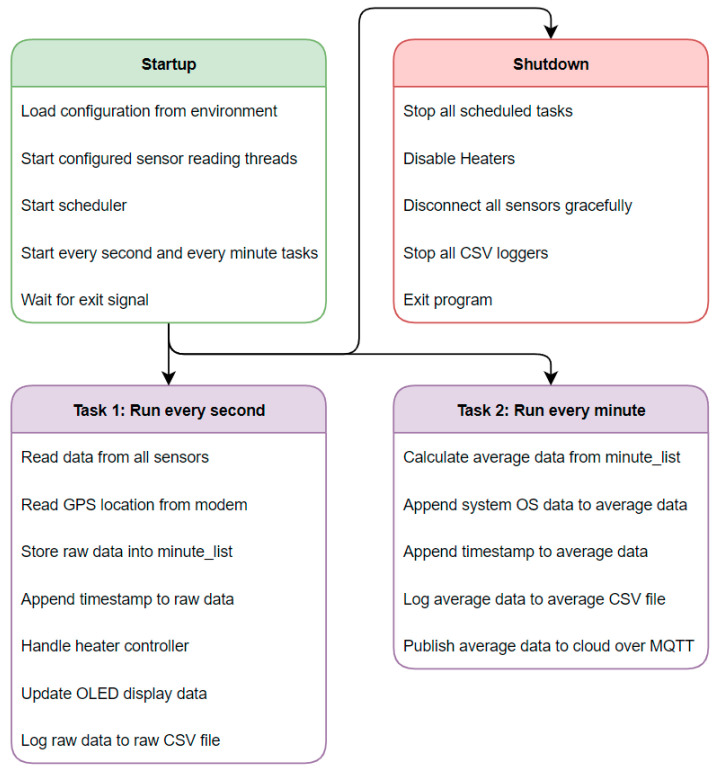
AQSN firmware flow diagram.

A basic outline of the Python program is given as a flowchart in Figure 6. Upon startup, the configuration is read in the form of Linux environment variables. This configuration specifies everything from which sensors are connected to sensor calibrations or cloud access variables. As a next step, two tasks are scheduled to run. Task 1 runs every second, and Task 2 every minute. This allows different actions to take place at different intervals.

#### 2.2.4. Dryer PID Controller

**Figure 7:**
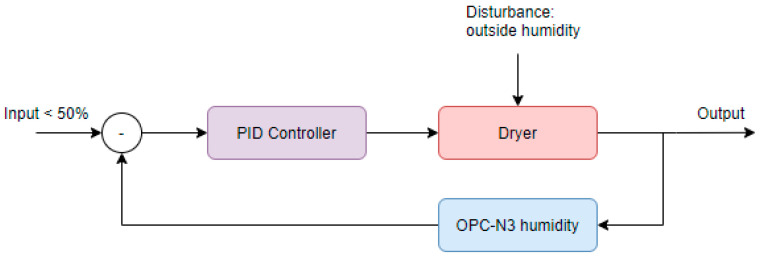
Dryer PID flow diagram.

As per the requirement that the system must be able to control the dehumidification process to constrain the temperature and humidity of the measured air, the firmware is also responsible for the dryer control. To achieve this, a PID controller was selected. Figure 7 shows the overview of the system.

While the dryer is part of the system whose operation is dependent on outside humidity conditions, the sensor of the OPC-N3 is used as the feedback value for the controller. As previously outlined, the measured relative humidity value is compared to the setpoint of 50% and fed into the PID controller as the error value. It has to be noted that this system only tries to accomplish a relative humidity below 50% at the sensor and does not interfere if this value is not exceeded. As both heaters have the same resistance, are powered at the same time with the same PWM duty cycle, and have the same airflow, the dehumidification process is expected to be approximately the same for both of them. This way, it is sufficient for the controller to only use the OPC-N3 relative humidity as an input and not measure the relative humidity of sample air at the gas sensors at all.

The PID controller parameters were estimated using the heuristic Ziegler–Nichols method [11]. This method allows the estimation of robust base parameters without a full system analysis. To start with this approach, the ultimate gain K_u_ and the corresponding period time T_u_ have to be determined. The first step is to set the K_i_ and K_d_ values to zero. Then K_p_ is increased in the running system until the output has stable oscillating behavior. As soon as this behavior can be observed, the ultimate gain K_u_ has been reached. Now, one can also determine the period T_u_ from the oscillations of the system output. Using this method, the best optimum settings of the dryer are K_p_ = 14, K_i_ = 0.005, and K_d_ = 0.

The hardware costs for one complete low-cost cloud-based AQSN was around 1500€ in material costs. A comparison of the developed system to the other low-cost air quality monitoring systems available on the market has already been discussed in detail in previous research proving the system to be cost-effective for both manufacturing and operation [9,10].

### 2.3. System Deployment

**Figure 8:**
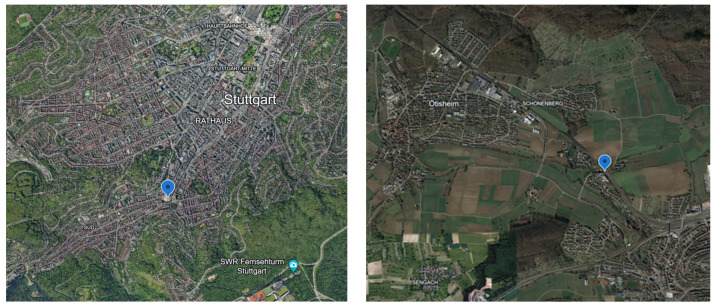
Measurement locations, (**left**): Marienplatz Stuttgart, (**right**): Ötisheim.

**Figure 9:**
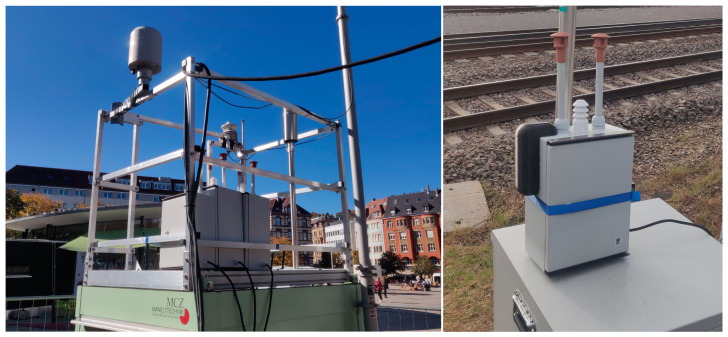
AQSN deployment, (**left**): Marienplatz Stuttgart, (**right**): Ötisheim.

After calibrating the AQSNs, all three were deployed at Marienplatz in Stuttgart starting on 22 October 2021 to check the operability of the system. There, a colocation of all three AQSNs was conducted. After three days, the AQSN or Airnode with the identification number 3 was moved to Ötisheim on 25 October 2021 while Airnodes 1 and 2 remained at Marienplatz. The reason for deploying 2 Airnodes in Marienplatz was to compare the Airnodes with one another as well for a longer period. All three AQSNs were retrieved on 25 November 2021. A picture of the ASQNs at Marienplatz (48.764037, 9.168815) and Ötisheim (48.957099, 8.831874) is given in Figure 8, and their respective locations in Figure 9. The two locations were chosen to investigate the AQSN behavior in an urban area (Marienplatz) and a regional background (Ötisheim). These locations were already equipped with high-end devices that were used to correlate the results from the AQSNs.

## 3. Results and Discussion

### 3.1. Quality Assurance

**Figure 10:**
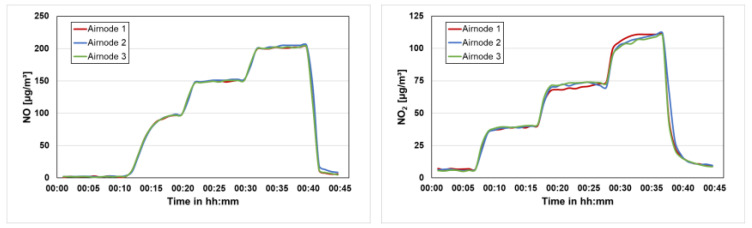
Quality assurance calibration for Airnodes 1, 2, and 3, (**left**): NO, (**right**): NO_2_.

Regular calibration is required for the sensors to maintain data quality during the measurement period. The AQSNs were calibrated in the laboratory before deployment for quality assurance. For the calibration of the gas sensors, all three constructed AQSNs were connected to a Serinus CAL 3000 gas phase titration (GPT) device. The GPT was programmed with a calibration sequence for multiple-point calibration. The concentrations for calibrating NO gas sensors were 0, 100, 150, and 200 ppb. For NO_2_ gas sensors, the calibration concentrations were 0, 50, 75, and 100 ppb. All three AQSNs were connected in parallel for the calibration. The resulting gas measurements are shown in Figure 10. The measurements from the three ASQNs 1, 2, and 3 are shown in red, blue, and green, respectively. From these measurements, the raw values were corrected using linear regression for each AQSN.

### 3.2. Prototype Performance

**Figure 11:**
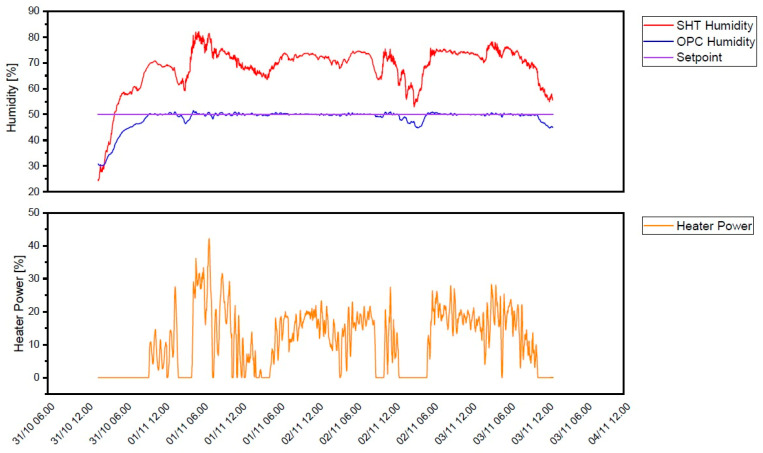
In-field dryer PID performance (red: ambient relative humidity in %; blue: relative humidity after drying measured at the OPC-N3 in %; yellow: PWM duty cycle of the heater in %).

With around a month of field operation and no major incidents, the developed AQSN system was proven to be robust. On average, 690 MB of cellular data volume per month was used by one AQSN. This was with frequent remote administration access and should be even lower in a long-term deployment scenario. The drying PI controller tuned via the heuristic Ziegler–Nichols method also appeared to be robust. An episode of three days with high relative humidity at Marienplatz can be seen in Figure 11. The ambient relative humidity (red-colored curve) rises above 50%, while the sample air at the OPC-N3 in blue was kept below the setpoint marked in purple. To dehumidify the sample air, only 40% of the maximum PWM duty cycle was necessary, which meant that the dryer performed well enough while still having some power reserves.

While developing this system, several problems occurred. One noteworthy problem was that of Electromagnetic Interference (EMI). The modem radiated a high amount of EMI inside the enclosure, which started inducing voltage in the analog lines of the gas sensors. The measurement accuracy was significantly improved by implementing grounded antistatic shielding around modem antenna pathways and all gas sensor analog lines.

The firmware for the AQSN developed during this research was capable of sensor modularity. This means any combination of sensors can be connected to a node and will, if configured accordingly within the environment variables, work seamlessly. That way, a node with, for example, only a PM sensor could easily be included in the sensor network.

### 3.3. Field Measurements

Starting on 25 October 2021, Airnodes 1 and 2 were deployed at Marienplatz while Airnode 3 was deployed at Ötisheim. Considering the ambient air temperature and relative humidity readings, the SHT30 sensor measured these values before the air was dehumidified. The air temperature and relative humidity sensor at OPC denoted measurements of these parameters at the OPC-N3 sensor after the sample air passed the dehumidification dryer. The comparative measurements with reference instruments were not performed for the complete period. Therefore, the reference instrument data were only presented for a certain time. There were no reference instruments at Ötisheim to measure O_3_ and CO. Hence, the comparison for the pollutants NO, NO_2_, and PM are presented.

**Figure 12:**
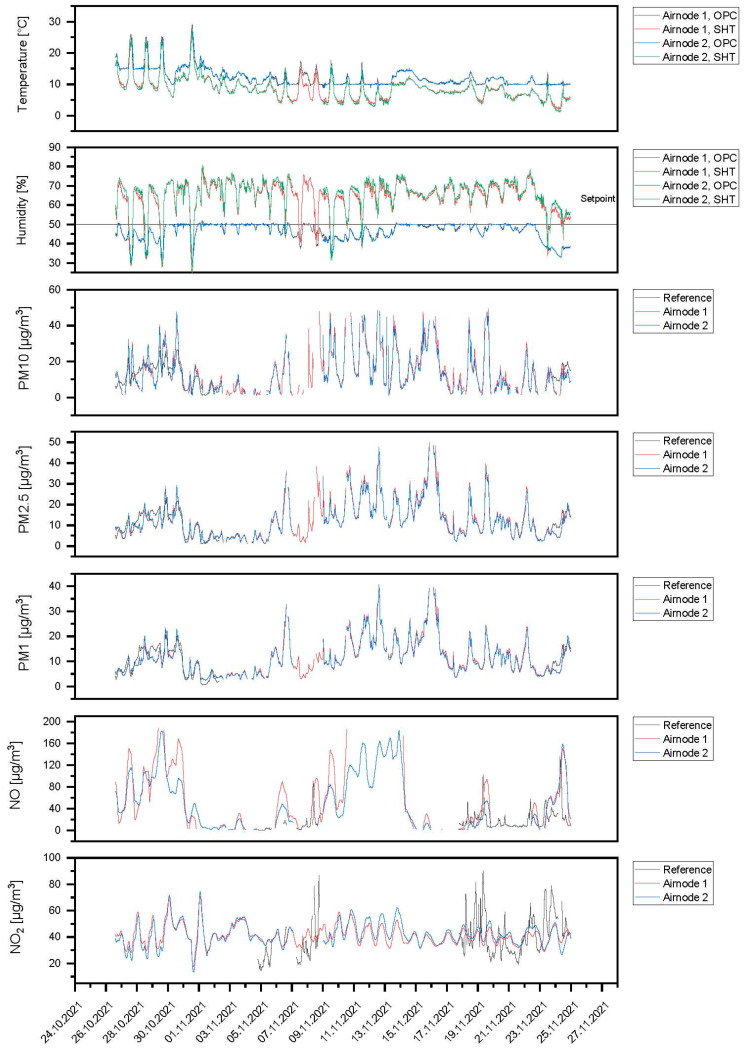
Field results for Airnode 1 and Airnode 2 at the Marienplatz Stuttgart location.

In Figure 12, the field results for Airnode 1 and Airnode 2 at the Marienplatz Stuttgart location are shown. Airnodes 1 and 2 were compared to the respective reference instruments (MLU 200A for NO and NO_2_; Grimm EDM180 for PM) for some time during the measurements at Marienplatz. The data measured by the sensors were corrected according to the reference instruments using linear regression. It was observed that the PM concentrations of both the Airnodes were in correlation with the reference instruments. The correlation values of PM10, PM2.5, and PM1 for Airnode 1 were 0.644, 0.839, and 0.906, respectively, and for Airnode 2 were 0.584, 0.829, and 0.9, respectively. The gas sensors also showed a good correlation with the reference instruments. From the results, it can be seen that the PM1 concentration at Marienplatz is relatively high, which shows that the PM concentration at Marienplatz is dominated by the fine fraction of PM. The high PM concentrations during the peak times of the day are visible during the weekdays indicating the traffic emissions at the measurement location. Relatively lower PM concentrations were observed on the weekends or during unstable atmospheric conditions. The gas pollutant concentrations varied following the reference instruments; however, differences in absolute concentrations were observed. There were some data losses due to technical issues during the measurement period.

**Figure 13:**
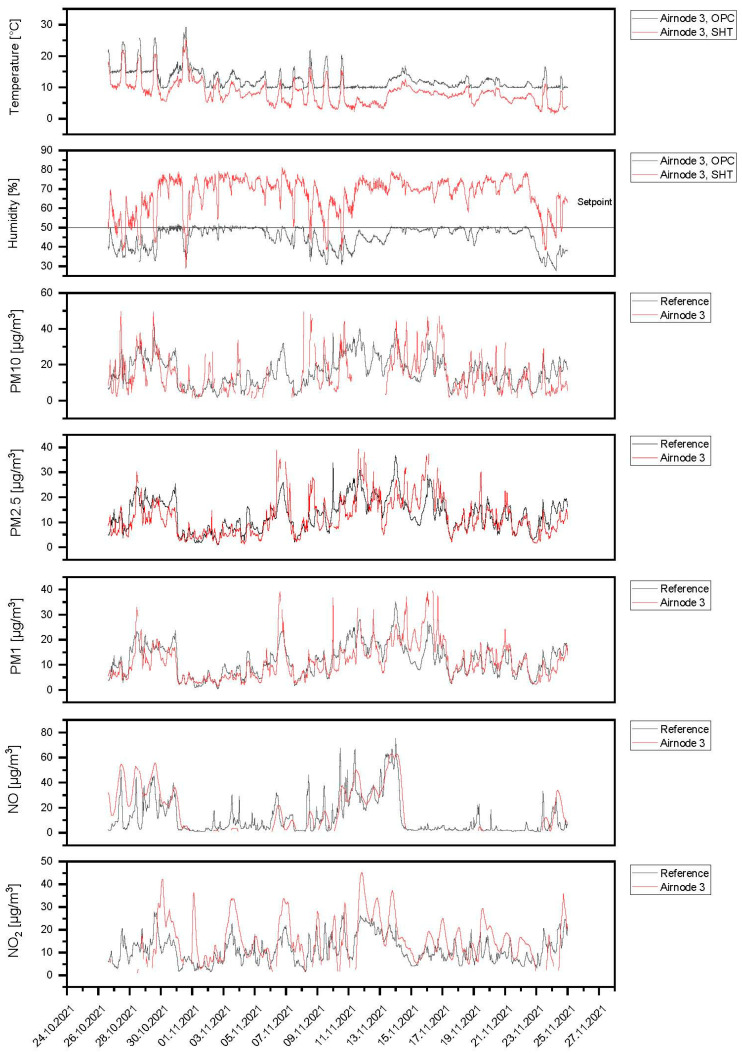
Field results for Airnode 3 at the Ötisheim location.

In Figure 13, the field results for Airnode 3 at the Ötisheim location are shown. Airnode 3 was compared and corrected to the reference instruments (Horiba APNA370 for NO and NO_2_; Grimm EDM180 for PM) as well as the Ötisheim measurement location. The correlation values for the PM fractions PM10, PM2.5, and PM1 were 0.549, 0.693, and 0.775, respectively. The correlation values for the gas sensors (NO and NO_2_) were 0.673 and 0.551. From the PM concentration results, it can be seen that the PM concentration trend measured at this location was different from the one at Marienplatz, mainly due to the source of PM emission being different at both locations. Nevertheless, the lower PM concentrations measured during the second week of measurements were also observed in Ötisheim as the PM concentration in the whole region was relatively low due to unstable weather conditions and rain events. Regarding the NO and NO_2_ low-cost gas sensor concentrations, it was observed that the concentration trend is similar to the one measured via the reference instrument, though the low-cost NO_2_ sensor is slightly sensitive to higher concentrations. The relatively high NO concentrations during the start, middle, and end of the measurement period were followed by the low-cost sensor relating to the reference instrument. 

To use such AQSNs on a larger scale, a scale-up in the production of Airnodes is an important aspect that should not be overlooked. The accuracy of the results depends on different factors such as meteorological conditions, mainly relative humidity and air temperature. The physical factors regarding the sensor itself like sensor drift, sensor life, etc. are important as well in this regard.

## 4. Conclusions and Outlook

The developed platform was capable of remotely measuring air quality and reporting that data to the cloud. The air pollutants such as PM, the gases NO, NO2, O3, and CO, as well as ambient air temperature and relative humidity were measured successfully. The chosen design considerations and the dryer optimization along with the improvement in mechanical and electronic systems proved to be helpful in the operation of the AQSNs.

This platform as described above has been developed and implemented during this research. Three AQSNs were constructed, calibrated, and validated in field deployments at Marienplatz and in Ötisheim for almost one month. During that time, the transmission of data into the cloud was robust. The implemented PI controller for dehumidifying the sample air with low-cost dryers proved to also be robust in the field tests.

The comparative measurements to the reference instruments at both locations showed a positive outcome for the AQSNs. The pollutant concentrations followed the trend concerning the reference instruments; however, the absolute concentrations varied now and then. The correlation of the AQSNs with the reference instruments revealed that the low-cost PM sensors correlated better to the respective reference instruments as compared to the low-cost gas sensors.

In this research, only a basic approach for gas sensor calibration was implemented. Many recent studies approach low-cost gas sensor calibration by employing machine learning methods such as random forest regression or multiple linear regression. Such an approach could be implemented with the AQSNs as a future research topic, as a lot of the processing power of the Raspberry Pi 4 remains unused with the current firmware. The latest patch in the AQSN firmware includes all raw OPC-N3 readings and raw gas sensor ADC readings in its JSON payload, which facilitates future work on this platform.

To reach truly autonomous operation, energy harvesting could be implemented in the future. A system generating solar power and storing it in a battery pack for continuous operation seems feasible. The biggest hurdle would probably be the excessive dryer power consumption resulting in a much bigger solar panel and battery size than necessary in a design without dryers.

Finally, the controller for the dryer has only been tuned using a heuristic approach. In the future, full system identification of the controlled system could improve the controller performance even further.

## Figures and Tables

**Table 1:** Comparison of computation systems.

	Weight	Microcontroller	Embedded System	Industrial Computer
**Processing power**	30%	1	4	5
**Hardware interfaces**	30%	5	4	2
**Power consumption**	20%	5	3	1
**Data storage**	20%	1	4	5
**Average score**		3	3.75	3.25
**Weighted score**		3	3.8	3.3

**Table 2:** Comparison of wireless communication systems.

	Weight	LoRaWAN	Sigfox	Cellular
**Data rate**	20%	2	1	4
**Range**	30%	3	3	4
**Localization**	10%	1	1	5
**Timekeeping**	10%	0	0	5
**Power consumption**	20%	5	5	1
**Operating expenses**	10%	5	2	1
**Average score**		2.7	2	3.4
**Weighted score**		2.9	2.4	3.3

## Data Availability

Data are contained within the article.
